# Plasma calcium, phosphorus, and calcium-to-phosphorus ratio as indicators of eggshell quality and reproductive performance in captive female Houbara bustards (<em>Chlamydotis undulata</em>)

**DOI:** 10.14202/vetworld.2026.2751-2762

**Published:** 2026-07-06

**Authors:** Sara M. Rashad

**Affiliations:** 1 Laboratory Manager, Bacteriology and Molecular Biology Units, International Foundation for Ecological Research (IFER), Errachidia, Morocco.

**Keywords:** calcium, captive breeding, conservation physiology, eggshell quality, Houbara bustard, mineral homeostasis, phosphorus, reproductive performance

## Abstract

**Background and Aim:**

The Houbara bustard (*Chlamydotis undulata*) is a vulnerable avian species whose conservation relies heavily on successful captive breeding. Eggshell abnormalities and egg retention are major reproductive constraints that can reduce breeding efficiency. Calcium (Ca) and phosphorus (P) play critical roles in eggshell formation and reproductive physiology; however, their relationship with reproductive performance in Houbara bustards has not been investigated. This study aimed to establish the first reproductive performance-based reference intervals for plasma Ca and P in captive female Houbara bustards, evaluate the calcium-to-phosphorus (Ca:P) ratio, and determine their associations with reproductive outcomes and eggshell abnormalities.

**Materials and Methods:**

A cross-sectional study was conducted on 239 adult female Houbara bustards aged 2–5 years. Blood samples were collected from 136 females during the breeding season and 103 females outside the breeding season. During the breeding season, birds were categorized as good layers (n = 70), soft-shelled egg producers (n = 26), retained egg (stuck egg) cases (n = 19), and non-laying females (n = 21). Plasma Ca and P concentrations were measured using an automated biochemistry analyzer, and the Ca:P ratio was calculated. Data were analyzed using descriptive statistics, the Kruskal–Wallis test, Dwass–Steel–Critchlow–Fligner post hoc comparisons, and Spearman correlation analysis.

**Results:**

Outside the breeding season, mean plasma Ca and P concentrations were 10.5 ± 0.69 mg/dL and 3.98 ± 0.94 mg/dL, respectively, with a mean Ca:P ratio of 2.78 ± 0.58. In good layers, reproductive season reference intervals were established as 10.8–27.8 mg/dL for Ca, 2.6–6.58 mg/dL for P, and 2.48–5.91 for the Ca:P ratio. Significant differences among reproductive groups were observed for Ca (χ² = 16.27, p < 0.001) and P (χ² = 11.12, p = 0.011), whereas the Ca:P ratio did not differ significantly (p = 0.657). Soft-shelled egg producers and retained egg cases exhibited significantly higher Ca concentrations than good layers, while P concentrations were significantly elevated in soft-shelled egg producers. Plasma Ca and P concentrations showed a strong positive correlation (r = 0.690, p < 0.001). Elevated Ca was also associated with soft-shelled eggs (r = 0.320, p = 0.001) and retained eggs (r = 0.240, p = 0.024).

**Conclusion:**

This study establishes the first reference intervals for plasma Ca and P based on reproductive performance in captive female Houbara bustards. Eggshell defects and egg retention were linked to altered mineral mobilization and utilization rather than Ca deficiency. Routine biochemical monitoring of Ca and P may serve as valuable biomarkers for reproductive assessment and improve breeding success in conservation programs for this vulnerable species.

## INTRODUCTION

The Houbara bustard (*Chlamydotis undulata*) is a seasonal breeding bird characterized by endocrine cycles extending from late winter through summer. Unlike continuously laying birds such as domestic hens, *C. undulata* exhibits pronounced reproductive-associated physiological fluctuations, making it an excellent model for investigating adaptive mineral mobilization and reproductive physiology. This species is of considerable conservation concern [1, 2] and is classified as Vulnerable on the International Union for Conservation of Nature Red List, with an estimated global population of approximately 49,000–62,000 individuals. Consequently, extensive captive breeding programs have been established in several countries to reduce the risk of extinction and support population reinforcement and reintroduction efforts. Despite these conservation initiatives, reproductive disorders, particularly eggshell abnormalities and egg retention, remain significant challenges that can compromise breeding success and reduce productivity in conservation breeding programs.

In laying birds, eggshell calcification imposes a substantial physiological demand on calcium (Ca) homeostasis because of the continuous requirement for ionized Ca during shell formation [3]. Ca is the principal structural component of the avian eggshell and is primarily deposited as Ca carbonate, whereas P plays essential roles in energy metabolism, skeletal maintenance, and mineral mobilization associated with eggshell formation [4, 6]. Dietary mineral imbalance, particularly excessive P relative to Ca, can impair Ca utilization and result in thinner, weaker eggshells that are more susceptible to cracking and structural defects [7, 8]. Therefore, assessment of reproductive performance is closely associated with circulating Ca and phosphorus (P) concentrations. In addition, the Ca:P ratio is considered an important indicator of mineral balance and endocrine regulation during eggshell formation in laying birds [5]. Recent evidence has further demonstrated that circadian and hormonal regulation of Ca and P metabolism is critical for synchronizing mineral availability with the oviposition cycle [3].

Limited information is available regarding the seasonal reproductive physiology of *C. undulata*, although previous studies have documented distinct patterns of breeding activity and associated endocrine changes during the laying period [9]. High-quality eggshells are essential for protecting the developing embryo during incubation and directly influence hatching success [10, 11]. This aspect is particularly important in *C. undulata*, where reproductive efficiency is a critical determinant of the success of conservation breeding programs [12]. Previous investigations have reported baseline hematologic and biochemical values in Houbara bustards [13], providing useful physiological reference data for health assessment and nutritional management.

Although baseline blood chemistry values have been established for Houbara bustards [13], existing studies have primarily focused on general physiological characterization rather than reproductive performance. To date, no study has investigated the relationship between plasma Ca and P concentrations, the Ca:P ratio, and specific reproductive outcomes in captive female Houbara bustards. Furthermore, the potential associations between mineral profiles and reproductive disorders such as soft-shelled eggs, retained eggs (stuck eggs), and non-laying status remain unknown. The absence of reproductive performance-based reference intervals for Ca and P represents a significant knowledge gap, limiting veterinarians' and conservation managers' ability to use biochemical indicators to early identify females at risk of reproductive failure. Addressing this gap is essential for improving reproductive monitoring, optimizing nutritional management, and enhancing breeding success in conservation programs for this vulnerable species.

We hypothesized that females producing defective eggs would exhibit altered plasma Ca, P, and Ca:P ratios compared with reproductively successful females. Therefore, this study aimed to establish the first reproductive performance-based reference intervals for plasma Ca and P concentrations in captive female Houbara bustards during and outside the breeding season. In addition, the study evaluated variations in the Ca:P ratio among females with different reproductive outcomes, including good layers, soft-shelled egg producers, retained egg cases, and non-laying females. Furthermore, the study investigated associations between plasma mineral profiles and eggshell abnormalities, including soft-shelled, stuck, and malformed eggs, and provided mechanistic insights into mineral homeostasis in a conservation-dependent avian species. Ultimately, the findings are expected to support evidence-based nutritional and management strategies that may reduce eggshell defects, improve reproductive performance, and strengthen the effectiveness of captive breeding programs for *C. undulata*.

## MATERIAL AND METHODS

### Ethical approval

The study was approved by the Institutional Animal Ethics Committee of the International Foundation for Ecological Research (IFER), Errachidia, Morocco, under approval number IFER-2025-01. All procedures were conducted in accordance with institutional animal welfare guidelines for the handling and management of captive avian species. Blood sampling was performed under the supervision of a licensed veterinarian using minimally invasive procedures to minimize stress, pain, and discomfort. Before sampling, all birds underwent clinical examination, and only clinically healthy adult female Houbara bustards were included. Birds were monitored throughout the study period to ensure their health and welfare, and all husbandry, restraint, and sampling procedures followed established avian welfare and captive breeding protocols.

### Study period and location

This study was conducted in 2025 (During breeding season, 15^th^ May to 15^th^ June; out of breeding season, 1^st^ December to 30^th^ December) at the IFER, Errachidia, Morocco. Sample collection was performed during and outside the breeding season in captive female Houbara bustards (*Chlamydotis undulata*). All birds were maintained under standardized husbandry conditions within the captive breeding facility.

### Study design

**Figure 1:**
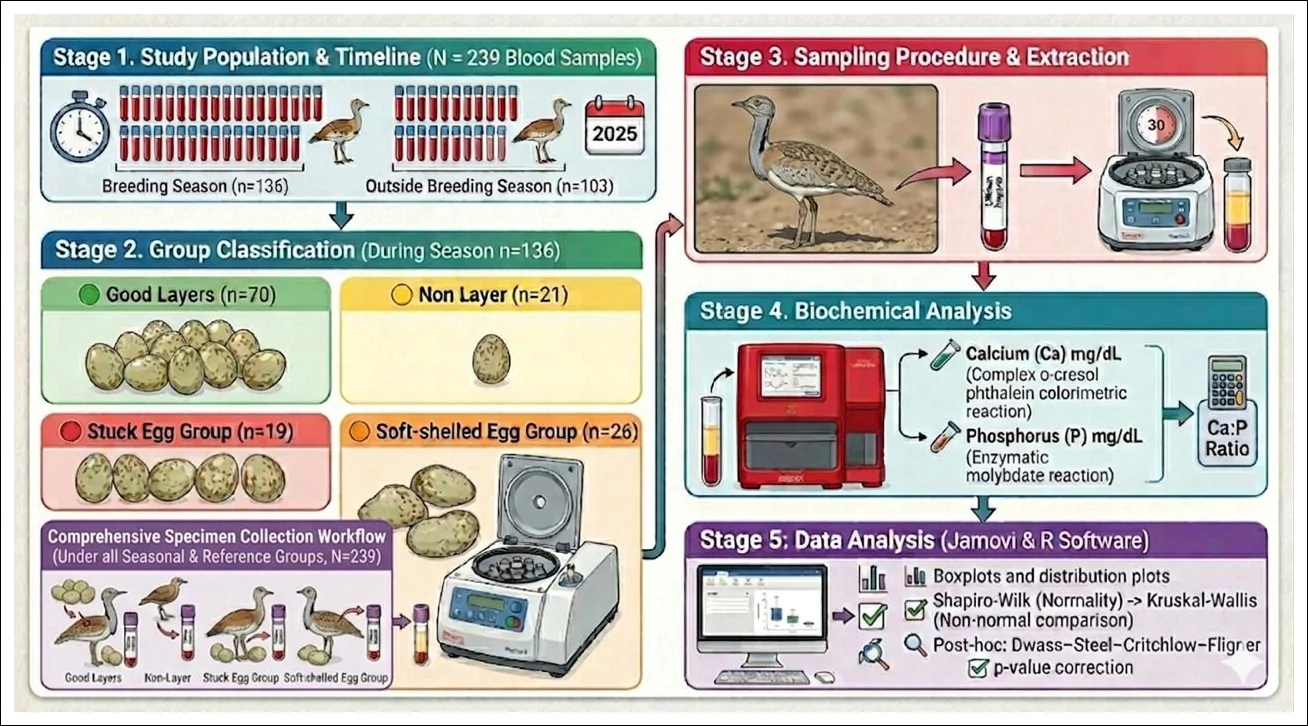
Schematic workflow of the experimental design, sample collection, biochemical profiling, and data analysis in breeding and non-breeding Houbara bustard females.

A cross-sectional study was conducted using 239 blood samples collected from captive female Houbara bustards aged 2–5 years. Of these, 136 samples were obtained during the breeding season, whereas 103 samples were collected outside the breeding season. Sampling was performed during the final third of the breeding season to evaluate mineral profiles in relation to reproductive performance and eggshell quality. The detailed experimental design, group classification, and methodological workflow are illustrated in Figure 1.

### Group classification

Birds sampled during the breeding season were classified into four groups according to reproductive performance and egg characteristics:

1. **Good layers (n = 70):** Females that produced normal eggs with regular laying intervals.

2. **Non-laying group (n = 21):** Females of optimal reproductive age that did not produce eggs during the breeding season.

3. **Retained egg (stuck egg) group (n = 19):** Females diagnosed with egg retention through physical examination and, when necessary, radiographic evaluation.

4. **Soft-shelled egg group (n = 26):** Females that produced soft-shelled eggs or eggs with shell abnormalities unsuitable for incubation.

Outside the breeding season, samples were collected from clinically healthy females (n = 103) and served as a reference group for comparison with birds during the breeding season.

### Sample collection

Blood samples were collected between 8:00 and 10:00 A.M. and within 30 min of capture. Sampling was performed during the latter part of the breeding season, specifically toward the end of the egg-laying period, to minimize disturbance to reproductive activity and reduce stress-related physiological variation. This timing enabled assessment of plasma Ca and P concentrations with minimal impact on egg production.

Before sampling, all birds underwent a clinical examination by a veterinarian and were confirmed to be clinically healthy. Approximately 3 mL of whole blood was collected from the brachial vein into lithium-heparinized blood collection tubes (Vacutest KIMA; KIMA, Arzergrande, Italy) following standard avian hematologic procedures [14]. Samples were subsequently centrifuged at 2,500 × *g* for 10 min using a bench-top centrifuge (Magafuge 8; Thermo Scientific, Waltham, MA, USA).

### Biochemical analysis

Plasma was separated immediately after centrifugation and analyzed for total Ca and P concentrations using a fully automated point-of-care biochemistry analyzer (Catalyst One; IDEXX Laboratories, Westbrook, ME, USA) as previously described for avian species [15, 16]. Subsequent analyses were performed using the Catalyst One system (IDEXX Laboratories).

Ca concentration was determined using a colorimetric method based on the formation of a colored complex with *o*-cresolphthalein in an alkaline medium. P concentration was measured enzymatically by reacting inorganic phosphate with ammonium molybdate and a reducing agent to produce a colored end product [17]. All samples were analyzed in duplicate to ensure analytical accuracy and reproducibility.

The Ca and P values obtained from the good layer group during the breeding season were used to establish reproductive performance-based reference intervals and to compare birds exhibiting eggshell abnormalities. The interpretation of biochemical variation and the establishment of reference intervals followed established veterinary clinical pathology guidelines [18, 19].

### Quality control

The Catalyst One system was routinely calibrated according to the manufacturer's instructions using IDEXX calibrators. Internal quality control procedures were performed throughout the study to ensure analytical reliability and consistency of results.

### Diet and management

All birds were maintained in open housing systems and exposed to natural environmental conditions; therefore, ambient temperature was not experimentally controlled. Each bird was housed individually, with one bird per enclosure. Birds received a balanced breeding diet formulated according to the nutritional requirements of captive Houbara bustards [20]. The diet contained 3.8% Ca and 0.75% P. Vitamin D supplementation was provided through a commercial vitamin–mineral premix incorporated into the diet according to breeding season recommendations.

Management practices were standardized across all study groups. Birds exhibiting clinical signs of disease or infection were excluded from the study to minimize potential confounding effects on biochemical measurements.

### Statistical analysis

The Ca:P ratio was calculated for each sample, and plasma Ca and P concentrations were expressed as mg/dL. Statistical analyses were performed using jamovi software (Version 2.6) [21], which is built on the R statistical environment [22]. 

Descriptive statistics, including mean, standard deviation, median, IQR, minimum, and maximum values, were calculated for each study variable. Data distribution and potential outliers were evaluated using boxplots and distribution plots. Normality was assessed using the Shapiro–Wilk test for all biochemical variables within each group. Based on the distribution of the data, appropriate statistical tests were selected. 

The Kruskal–Wallis test was used to analyze non-normally distributed variables. When significant differences were detected, post hoc pairwise comparisons were performed using the Dwass–Steel–Critchlow–Fligner test with adjusted p-values to control the type I error rate associated with multiple comparisons [23]. Statistical significance was established at p < 0.05.

## RESULTS

### Plasma Ca, P, and Ca:P ratio outside the breeding season

**Figure 2:**
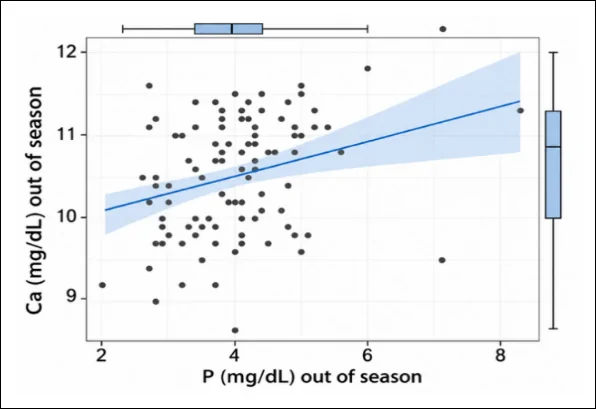
Scatterplot displaying the relationship between plasma calcium (Ca; mg/dL) and phosphorus (P; mg/dL) concentrations in adult female Houbara bustards outside the egg-laying season (n = 103). Marginal boxplots illustrate the distribution of each mineral, with boxes representing the IQR, horizontal lines indicating the median, whiskers representing the minimum and maximum, and individual dots indicating outliers.

Adult female Houbara bustards outside the egg production season (n = 103) exhibited a median plasma Ca concentration of 10.7 mg/dL, a median plasma P concentration of 4.0 mg/dL, and a median Ca:P ratio of 2.68. Plasma Ca concentrations ranged from 8.6 to 11.8 mg/dL, whereas plasma P concentrations ranged from 2.0 to 8.3 mg/dL. These findings indicate a relatively stable mineral profile outside the reproductive season and provide valuable baseline reference values for comparison with birds during the breeding season. The observed stability in mineral homeostasis may serve as a useful physiological benchmark for health assessment, nutritional management, and reproductive monitoring in captive female Houbara bustards (Table 1). Spearman correlation analysis demonstrated a significant positive correlation between plasma Ca and P concentrations (r = 0.315, p < 0.001) (Figure 2).

### Comparison of plasma Ca, P, and Ca:P ratio among reproductive groups

**Figure 3:**
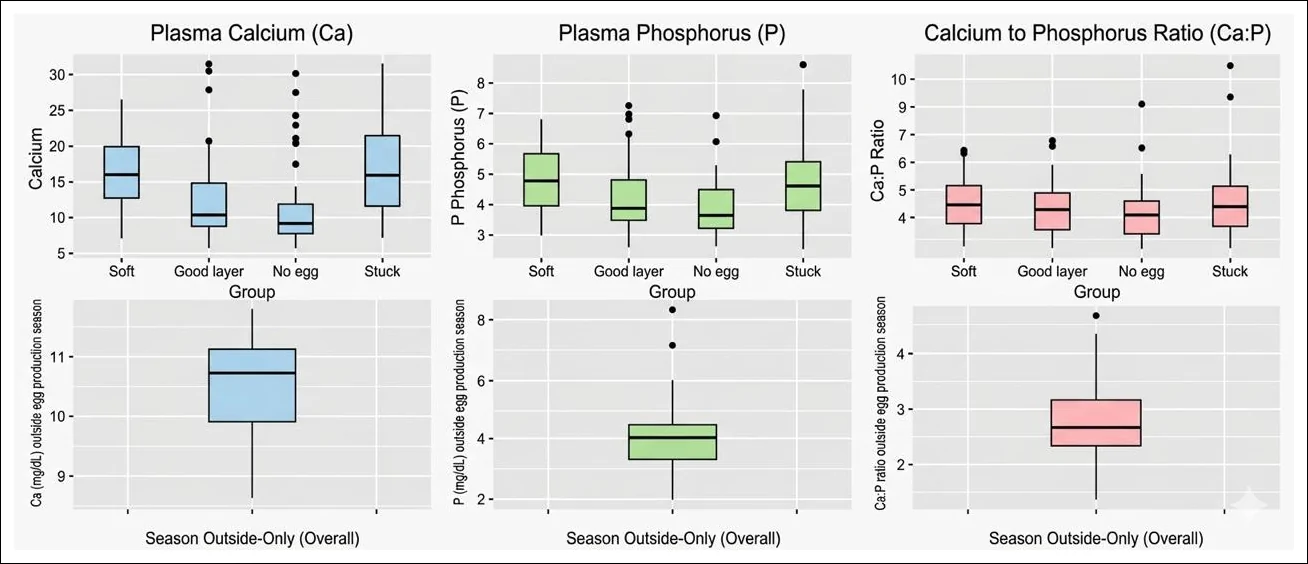
Boxplots representing the distribution of plasma Ca, P, and Ca:P ratio in adult female Houbara bustards outside the egg production season (n = 103) and during the breeding season across four reproductive groups (n = 136). The boxes represent the IQR, horizontal lines indicate median values, whiskers indicate minimum and maximum values, and dots indicate outliers. Ca = Calcium, P = Phosphorus.

Descriptive statistics for plasma Ca, P, and Ca:P ratio in the good layer, soft-shelled egg, retained egg (stuck egg), and non-laying groups are summarized in Table 2. Higher mean plasma Ca concentrations were observed in the soft-shelled egg and retained egg groups compared with the good layer and non-laying groups. The retained egg group also exhibited the highest mean P concentration and Ca:P ratio. Greater variability in the Ca:P ratio was observed in the retained egg and non-laying groups, as reflected by larger SD values and wider concentration ranges. Median values and IQRs demonstrated higher plasma Ca concentrations in the soft-shelled egg and retained egg groups than in the other groups. The highest plasma P concentrations were observed in the retained egg group. The Ca:P ratio exhibited greater variability and a wider distribution in the retained egg and non-laying groups (Figure 3).

**Table 1:** Plasma Ca, P, and Ca:P ratio in adult female Houbara bustards outside the breeding season.

**Variable**	**Mean ± SD (mg/dL)**	**Median [IQR] (mg/dL)**	**Min–Max (mg/dL)**	**Normality p-value**
Ca	10.5 ± 0.695	10.7	8.6–11.8	0.006**
P	3.98 ± 0.945	4.0	2.0–8.3	<0.001***
Ca:P ratio	2.78 ± 0.58	2.68	1.36–4.6	0.013*

Parametric analysis: Mean ± SD, Non-parametric analysis: Median [IQR], p < 0.05, ** p < 0.01, *** p < 0.001. Ca = Calcium, P = Phosphorus.

**Table 2:** Descriptive statistics of plasma Ca, P, and Ca:P ratio among reproductive groups during the breeding season.

**Group**	**Variable**	**Mean ± SD ** **(mg/dL)**	**Median [IQR] ** **(mg/dL)**	**Min–Max ** **(mg/dL)**	**Normality ** **p-value**	**Suggested analysis**
Good layers (n = 70)	Ca	15.9 ± 5.39	13.4 (12.0–18.4)	7.2–31.3	<0.001	Non-parametric
	P	4.31 ± 1.14	3.9 (3.6–4.8)	2.1–7.1	0.002	Non-parametric
	Ca:P	3.72 ± 0.859	3.56 (3.1–4.12)	2.38–6.27	0.001	Non-parametric
Soft-shelled egg group (n = 26)	Ca	19.0 ± 4.73	18.4 (15.5–21.8)	11.5–27.5	0.322	Parametric
	P	5.0 ± 1.38	4.6 (3.95–6.27)	2.8–7.5	0.165	Parametric
	Ca:P	3.89 ± 0.746	3.72 (3.38–4.32)	2.8–5.85	0.061	Borderline normality
Non-laying group (n = 21)	Ca	15.0 ± 5.63	12.7 (11.7–13.9)	10.9–30.3	<0.001	Non-parametric
	P	3.94 ± 1.11	3.7 (3.3–4.1)	2.5–6.9	<0.001	Non-parametric
	Ca:P	3.87 ± 1.23	3.46 (3.16–4.04)	2.66–8.19	<0.001	Non-parametric
Retained egg group (n = 19)	Ca	19.3 ± 5.8	18.8 (14.4–23.3)	11.1–30.9	0.428	Parametric
	P	4.92 ± 1.75	4.9 (3.85–5.75)	1.9–8.8	0.226	Parametric
	Ca:P	4.23 ± 1.76	3.74 (3.09–4.57)	2.52–10.3	<0.001	Non-parametric

Parametric analysis: Mean ± SD, Non-parametric analysis: Median [IQR], Borderline normality: May require data transformation or parametric analysis with caution. p < 0.05, ** p < 0.01, *** p < 0.001. Ca = Calcium, P = Phosphorus.

### Reference intervals for plasma Ca, P, and Ca:P ratio in good layers

Reference intervals for plasma Ca, P, and Ca:P ratio were established using the good layer group, as summarized in Table 3. The intervals were calculated using the non-parametric 2.5th–97.5th percentile method and may serve as reproductive performance-based reference values for captive female Houbara bustards.

### Differences among reproductive groups

The Kruskal–Wallis test revealed significant differences among reproductive groups for plasma Ca (χ² = 16.27, p < 0.001) and plasma P (χ² = 11.12, p = 0.011). In contrast, the Ca:P ratio did not differ significantly among groups (χ² = 1.61, p = 0.657) (Table 4).

### Post hoc pairwise comparisons

**Figure 4:**
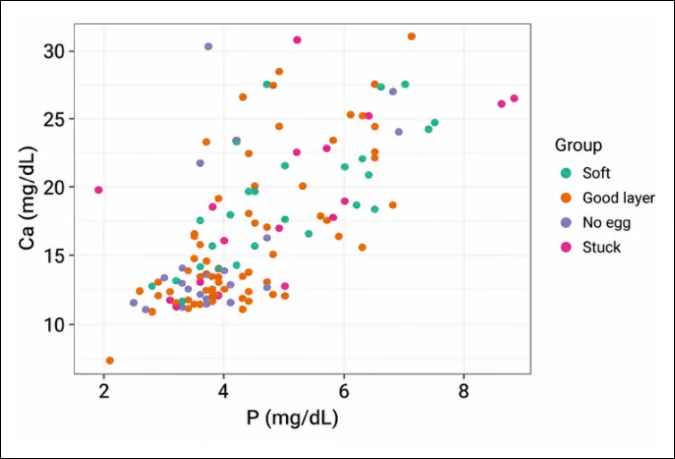
Scatterplot illustrating the relationship between plasma calcium (Ca) and phosphorus (P) concentrations during the breeding season in captive female Houbara bustards (n = 136). Each point represents an individual bird. Colors indicate reproductive groups, including good layers, soft-shelled egg producers, retained egg cases, and non-laying females.

Dwass–Steel–Critchlow–Fligner post hoc analysis confirmed the Kruskal–Wallis results. Plasma Ca concentrations were significantly higher in both the soft-shelled egg and retained egg groups than in the good layer group. Plasma P concentrations were significantly higher in the soft-shelled egg group than in the good layer group (Table 5). Higher plasma Ca and P concentrations were generally observed in the soft-shelled egg and retained egg groups than in the good layer group (Figure 4).

**Table 3:** Reproductive performance-based reference intervals for plasma Ca, P, and Ca:P ratio in good layer female Houbara bustards.

**Parameter**	**Median (50** **^th^** ** percentile) (mg/dL)**	**Reference interval (2.5** **^th^** **–97.5** **^th^** ** percentile) (mg/dL)**
Ca	13.4	10.8–27.8
P	3.9	2.6–6.58
Ca:P	3.56	2.48–5.91

Ca = Calcium, P = Phosphorus.

**Table 4:** Kruskal–Wallis analysis of plasma Ca, P, and Ca:P ratio among reproductive groups.

**Variable**	**χ²**	**p-value**
Ca	16.27	<0.001***
P	11.12	0.011**
Ca:P	1.61	0.657 (NS)

Significant differences were observed for both Ca and P. p < 0.05, ** p < 0.01, *** p < 0.001, NS = non-significant (p > 0.05). Ca = Calcium, P = Phosphorus.

**Table 5:** Dwass–Steel–Critchlow–Fligner post hoc pairwise comparisons for plasma Ca and P.

**Variable**	**Pairwise comparison**	**W**	**p-value**
Ca	Soft-shelled egg vs. good layer	4.41	0.002**
	Retained egg vs. good layer	3.18	0.025*
	Non-laying vs. good layer	−1.48	0.296 (NS)
P	Soft-shelled egg vs. good layer	3.16	0.025*
	Retained egg vs. good layer	2.08	0.141 (NS)
	Non-laying vs. good layer	−2.31	0.103 (NS)

The post hoc analysis confirmed significant differences for both Ca and P. p < 0.05, ** p < 0.01, *** p < 0.001, NS = non-significant (p > 0.05). Ca = Calcium, P = Phosphorus.

### Correlation analysis

Spearman correlation analysis demonstrated a significant positive correlation between plasma Ca and P concentrations (r = 0.690, p < 0.001) (Table 6). A moderate positive correlation was observed between plasma Ca concentration and the occurrence of soft-shelled eggs (r = 0.320, p = 0.001), whereas plasma P concentration exhibited a weak but significant positive correlation with soft-shelled egg production (r = 0.229, p = 0.025). Plasma Ca concentration also showed a weak but significant positive correlation with retained egg occurrence (r = 0.240, p = 0.024). No significant correlations were detected between mineral concentrations and the non-laying group.

These findings suggest that Ca may play a more prominent role than P in reproductive abnormalities, particularly in soft-shelled egg production and egg retention, although the observed associations were weak to moderate.

**Table 6:** Spearman correlation analysis of plasma mineral profiles and reproductive outcomes.

**Variables**	**Spearman's rho (r)**	**p-value**	**Interpretation**
Ca vs. P	0.690	<0.001	Significant positive correlation
Ca vs. soft-shelled eggs	0.320	0.001	Moderate significant positive correlation
P vs. soft-shelled eggs	0.229	0.025	Weak significant positive correlation
Ca vs. non-laying status	−0.110	0.298	No significant correlation
P vs. non-laying status	−0.172	0.103	No significant correlation
Ca vs. retained eggs	0.240	0.024	Weak significant positive correlation
P vs. retained eggs	0.157	0.142	No significant correlation

p < 0.05, ** p < 0.01, *** p < 0.001. Ca = Calcium, P = Phosphorus.

## DISCUSSION

### Novelty and significance

This study provides the first comprehensive evaluation of reproductive performance by quantifying plasma Ca, P, and the Ca:P ratio in captive female Houbara bustards (*Chlamydotis undulata*). Unlike previous studies that were limited to general biochemical characterization, the present work directly links mineral homeostasis with reproductive outcomes, including soft-shelled eggs and retained (stuck) eggs. The findings suggest that eggshell abnormalities are associated with impaired mineral utilization and deposition efficiency rather than simple mineral deficiency. Consequently, this study provides a mechanistic framework with direct relevance to reproductive monitoring, nutritional management, and conservation breeding of endangered avian species.

### *C. undulata* as a model for seasonal Ca homeostasis in conservation-dependent birds

*C. undulata* is a valuable model for understanding seasonal Ca and P homeostasis due to its distinct endocrine cycles. Unlike continuously laying birds such as domestic hens, it exhibits pronounced physiological transitions between reproductive and non-reproductive periods, enabling investigation of adaptive mineral mobilization associated with egg production. These characteristics are particularly relevant to conservation breeding programs, including those conducted at IFER, where eggshell quality directly influences reproductive success and hatchability.

Establishing associations between plasma mineral status and reproductive outcomes may help reduce embryo loss and improve breeding efficiency. Furthermore, the findings have practical implications for both captive management and potential *in situ* conservation strategies. The relatively large dataset used in this study (n = 239, including 136 breeding season birds) further strengthens the utility of the *C. undulata* as a model species for conservation physiology and reproductive biology.

### Mechanistic basis of mineral regulation during eggshell formation

**Figure 5:**
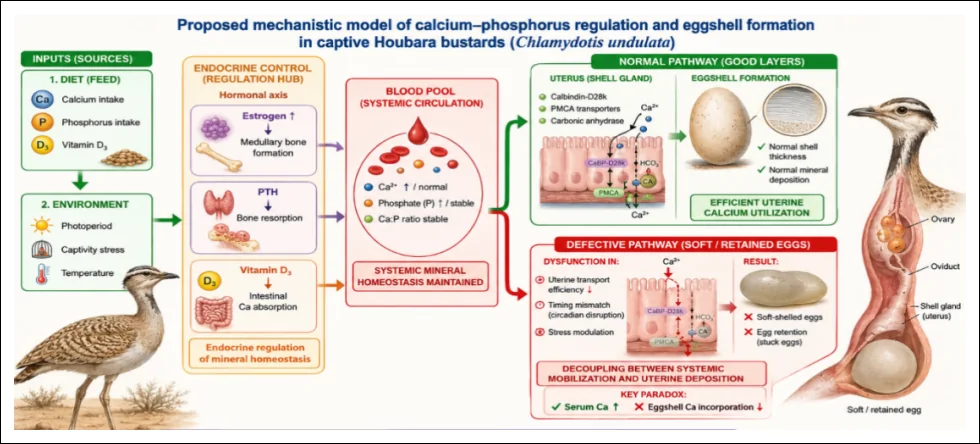
Conceptual model describing calcium (Ca) and phosphorus (P) regulation during eggshell formation in captive Houbara bustards. These pathways influence medullary bone mobilization and intestinal Ca absorption. In high-performing layers, coordinated endocrine signaling promotes efficient uterine Ca transport through calbindin and plasma membrane Ca ATPase (PMCA) pathways, resulting in normal eggshell formation. In contrast, soft-shelled egg and retained egg cases may involve a functional dissociation between systemic Ca mobilization and uterine deposition efficiency, leading to elevated circulating Ca concentrations but impaired eggshell mineralization. This conceptual framework suggests that eggshell abnormalities are more closely associated with utilization inefficiency than with Ca deficiency.

In avian species, Ca homeostasis during egg formation is tightly regulated through integrated endocrine and cellular mechanisms involving estrogen, PTH, and vitamin D₃ metabolites (Figure 5). Estrogen stimulates the formation of medullary bone, which serves as a readily mobilizable Ca reserve, whereas 1,25-dihydroxyvitamin D₃ enhances intestinal Ca absorption by increasing the expression and activity of Ca-binding proteins such as calbindin-D28k and PMCA transporters in enterocytes and uterine epithelial cells [4, 24].

During eggshell formation, Ca is actively transported across the uterine epithelium into the shell gland lumen through transcellular pathways [25]. This process is energy-dependent and synchronized with circadian mechanisms regulating oviposition. Any impairment in epithelial transporter function or uterine glandular activity may result in a mismatch between systemic Ca availability and eggshell deposition, thereby producing eggshell defects despite adequate or elevated circulating Ca concentrations [26].

In the present study, elevated plasma Ca concentrations in birds producing defective eggs likely reflect sustained medullary bone mobilization under preserved endocrine stimulation but reduced efficiency of uterine incorporation and shell mineralization.

### Seasonal variation in plasma Ca and P

Outside the laying season, plasma Ca and P concentrations were consistent with previously reported reference intervals for adult Houbara bustards [13]. Bailey *et al.* [12] also reported age-related variations in biochemical parameters among chicks, juveniles, and adults, indicating an association between mineral status and physiological development. The baseline values observed in the present study likely reflect the endocrine quiescence characteristic of seasonal breeders [9], during which Ca homeostasis depends primarily on dietary absorption and skeletal equilibrium, in the absence of the substantial mineral demands of eggshell formation. The relatively narrow variation observed supports the stability of mineral regulation during the non-reproductive period.

In contrast, the laying season was associated with marked increases in circulating Ca concentrations, particularly in females that produced soft-shelled eggs or experienced egg retention. Hypercalcemia during the laying period represents a normal physiological adaptation and is primarily mediated by estrogen-induced medullary bone formation and enhanced intestinal Ca absorption [4, 27]. These mechanisms facilitate rapid mobilization of ionized Ca to meet the substantial demands of shell calcification. Therefore, the elevated Ca concentrations observed in laying Houbara bustards are consistent with physiological adaptation rather than pathological disturbance, in agreement with observations reported in other avian species [28].

### Mineral changes associated with eggshell defects

Eggshell abnormalities contribute indirectly to reproductive failure because defective eggs are often unsuitable for incubation and may result in embryo loss.

One of the most notable findings of this study was that females producing soft-shelled eggs and females experiencing egg retention exhibited significantly higher plasma Ca concentrations than good layers. This observation challenges the traditional assumption that eggshell abnormalities primarily result from Ca deficiency. Previous studies in commercial laying hens have similarly reported eggshell defects in the presence of hypercalcemia when mineral deposition efficiency within the shell gland is impaired [29, 30].

Eggshell calcification is a highly regulated and time-dependent process occurring within the uterine portion of the oviduct. Successful shell formation depends not only on systemic availability of Ca and P but also on efficient epithelial transport, matrix protein secretion, carbonic anhydrase activity, and precise circadian regulation [3, 31, 32]. Consequently, elevated plasma Ca concentrations in females producing defective eggs may indicate intensified skeletal mobilization exceeding local deposition capacity. Under such circumstances, systemic mobilization remains active, whereas uterine incorporation becomes inefficient, leading to transient accumulation of Ca within the circulation.

This dissociation between mineral mobilization and deposition may result from subtle alterations in uterine physiology, endocrine timing, or stress-related modulation under captive conditions. Although direct assessment of ionized Ca, estrogen concentrations, and uterine transporter expression was beyond the scope of this study, the observed patterns are consistent with reduced deposition efficiency despite preserved systemic mobilization [33, 34].

Plasma P concentrations further support this interpretation. Although group differences were less pronounced than those observed for Ca, females producing soft-shelled eggs exhibited significantly higher plasma P concentrations than good layers. P plays a critical role in bone remodeling and mineral homeostasis [6]. During enhanced mobilization of medullary bone reserves, P is released simultaneously with Ca, reflecting increased skeletal turnover [24, 34, 35]. Therefore, elevated plasma P concentrations may represent a secondary consequence of intensified mineral mobilization rather than an independent pathological process.

### Stability of the Ca:P ratio and physiological regulation

Despite significant differences in absolute Ca and P concentrations among reproductive groups, the Ca:P ratio remained relatively stable. In avian species, Ca and P homeostasis is coordinated through integrated endocrine regulation involving PTH, estrogen, and vitamin D metabolites [4, 27]. Maintenance of a relatively stable Ca:P ratio suggests that systemic regulatory mechanisms remain functional despite fluctuations in individual mineral concentrations.

Therefore, the present findings indicate quantitative shifts in circulating mineral concentrations within an otherwise preserved physiological regulatory framework rather than evidence of generalized mineral imbalance. Furthermore, egg retention may prolong estrogenic stimulation and skeletal Ca mobilization beyond the normal period of shell deposition. Continued mobilization without successful oviposition may contribute to sustained elevations in circulating Ca concentrations. Although the precise mechanisms require further investigation, this interpretation is consistent with established models of avian reproductive endocrinology.

### Conservation and biological significance

From a conservation perspective, reproductive success in captive breeding programs is influenced by numerous interacting physiological, nutritional, environmental, and infectious factors. Diseases, including *E. coli* infections [36], together with eggshell abnormalities and egg retention, represent important obstacles to successful reproduction in captive *C. undulata*.

The observed associations between plasma mineral profiles and reproductive outcomes indicate that biochemical monitoring may provide a useful approach for the early identification of females at risk of reproductive failure. Routine assessment of plasma Ca and P concentrations could therefore complement traditional reproductive evaluations and assist in optimizing nutritional and management strategies.

These findings highlight the value of integrating physiological monitoring with reproductive assessment in conservation breeding programs. Such an approach may not only improve productivity in captive populations but also support long-term *ex situ* conservation efforts for this vulnerable species.

## CONCLUSION

This study established the first reproductive performance-based reference intervals for plasma Ca, P, and Ca:P ratio in captive female *C. undulata* and demonstrated significant associations between mineral profiles and reproductive outcomes. Outside the breeding season, females exhibited relatively stable plasma mineral concentrations, whereas breeding season birds showed marked increases in circulating Ca and P. Reproductive performance-based reference intervals were established for good layers at 10.8–27.8 mg/dL for Ca, 2.6–6.58 mg/dL for P, and 2.48–5.91 for the Ca:P ratio. Females producing soft-shelled eggs and those with retained eggs exhibited significantly higher plasma Ca concentrations than good layers, while plasma P concentrations were significantly elevated in soft-shelled egg producers. A strong positive correlation was observed between plasma Ca and P concentrations (r = 0.690, p < 0.001), further supporting the coordinated regulation of mineral homeostasis during reproduction.

Importantly, the findings indicate that eggshell abnormalities and egg retention are more likely associated with impaired mineral utilization and uterine deposition efficiency than with absolute mineral deficiency. The absence of significant differences in the Ca:P ratio among reproductive groups suggests that systemic endocrine regulation of mineral balance remains largely intact despite alterations in circulating mineral concentrations. These results provide new mechanistic insights into reproductive physiology and mineral metabolism in a conservation-dependent avian species.

From a practical perspective, routine monitoring of plasma Ca and P concentrations may serve as a valuable tool for reproductive assessment and early identification of females at risk of eggshell defects or egg retention. Integration of biochemical monitoring into captive breeding programs could facilitate evidence-based nutritional and management interventions to improve reproductive success and reduce egg losses.

A major strength of this study is the relatively large sample size, including 239 females and 136 breeding season birds, which enabled the establishment of reproductive performance-based reference intervals and the evaluation of mineral profiles across distinct reproductive categories. However, the study was limited by its cross-sectional design and the lack of measurements of ionized Ca, vitamin D metabolites, reproductive hormones, and uterine transporter activity, which may further clarify the mechanisms underlying eggshell abnormalities.

Future studies should incorporate longitudinal monitoring throughout the laying cycle, evaluate endocrine and molecular regulators of mineral transport, and investigate the relationships among nutrition, mineral metabolism, and reproductive performance under both captive and wild conditions. Such investigations would provide a more comprehensive understanding of mineral regulation and reproductive physiology in Houbara bustards.

Overall, this study demonstrates that plasma Ca and P concentrations are useful biomarkers of reproductive status in captive female Houbara bustards and provides a foundation for improving reproductive monitoring, nutritional management, and conservation breeding strategies for this vulnerable species.

## DATA AVAILABILITY

The data generated during the study are included in the manuscript.

## GENERATIVE AI DECLARATION

The author used generative artificial intelligence (AI) tools to improve the language, readability, and presentation of the manuscript, as well as to enhance the quality and formatting of figures. All scientific content, data accuracy, and final manuscript revisions were reviewed and approved by the author, who take full responsibility for the content of this article.

## AUTHOR’S CONTRIBUTIONS

**SMR:** Conception and design of the study, data collection, analysis and interpretation, manuscript writing, and approval of the final version of the manuscript.
